# Molecular Diagnosis of Panel-Based Next-Generation Sequencing Approach and Clinical Symptoms in Patients With Glycogen Storage Disease: A Single Center Retrospective Study

**DOI:** 10.3389/fped.2020.600446

**Published:** 2020-12-03

**Authors:** Shen Ying, Zhang Zhihua, Zheng Yucan, Jin Yu, Lin Qian, Zheng Bixia, Cheng Weixia, Liu Zhifeng

**Affiliations:** Department of Gastroenterology, Children's Hospital of Nanjing Medical University, Nanjing, China

**Keywords:** glycogen storage disease, next-generating sequencing, panel, variants, uncooked cornstarch

## Abstract

**Aim:** The aim of this study was to investigate the clinical utility of panel-based next-generation sequencing (NGS) in the diagnostic approach of glycogen storage disease (GSD).

**Methods:** We performed a retrospective review of the 32 cases with suspected GSDs between April 2013 and November 2019 through panel-based NGS, clinical and biochemical data and long-term complications.

**Results:** Of the 32 clinical cases, we identified 41 different variants, including 24 missense (58.5%), one synonymous (2.4%), three nonsense (8%), one splice (2.4%), four frameshift (9.8%), one deletion (2.4%), four insertions (9.8%), two deletion-insertion (4.9%) and one duplication(2.4%), of which 13(31.7%) were previously unreported in the literature. In addition, patients with different types of GSDs showed important differences in biochemical parameters (i.e., CK, rGGT, TG, and UA).

**Conclusions:** The panel-based NGS played an important diagnostic role in the suspicious GSDs patients, especially in the mild phenotype and ruled out detectable pathologic conditions. Besides, differences between our GSDs patients reflect biochemical heterogeneity.

Glycogen is a branched glucose polymer found primarily in the cytoplasm of liver and muscle cells (1). While liver glycogen primarily maintains blood glucose concentrations on demand, skeletal muscle glycogen typically caters to energy requirement after high-intensity activity. Glycogen storage disease (GSD) is an inherited metabolic disease caused by inadequacy of various enzymes that control the synthesis, regulation, and degradation of glycogen (2). Basis the underlying enzyme deficiency, GSD is classified into >14 types (3). Liver GSDs, including GSD 0, Ia, Ib, III, IV, VI, IX, and X, cause growth failure and hepatomegaly during fasting, whereas muscle GSDs, including GSD II, V, and VII, cause exercise intolerance, cramping, and cardiomyopathy (4). These inherited diseases vary widely in age of onset, morbidity, and mortality.

Cumulatively, incidence of GSDs is rare [~1 child per 69,054 births (5)]; however, they occur across ethnicities with the age of onset ranging from fetal age to adulthood. GSDs exhibit autosomal recessive inheritance except type IX and Danon disease (X-linked recessive). Depending on the enzyme mutation, some patients with GSDs die within the first year of life, whereas have a favorable prognosis (6). While GSDs are clinically distinguishable, specific biomarkers are lacking. With molecular diagnosis of peripheral blood, the precise GSD type and patient prognosis can be ascertained. Early diagnosis and treatment are important in improving the patient's quality of life.

Full-gene Sanger sequencing has remained the gold standard for several years. However, it only allows assessing one gene by gene or exon at a time, making it time-consuming and effort intensive (7). High-throughput sequencing techniques that sequence all exons at once, can for accurate and effective diagnosis, while reducing costs and time. Target enrichment–based NGS is more efficient in testing large numbers of patients with similar symptoms and with multiple known candidate variants of genes than whole-genome sequencing. This study aimed to evaluate the clinical and genetic analyses, and their application as research tools for panel-based NGS analysis in GSDs to understand the possible genotype-phenotype correlation. Based on the findings, we establish the feasibility of this NGS-based genetic diagnosis method for GSD patients.

## Materials and Methods

### Patients and DNA Samples

Blood samples from 32 patients of GSD between April 2013 and November 2019 were sent to the Nanjing Key Laboratory of Pediatrics at the Children's Hospital of Nanjing Medical University in Nanjing, China, for genetic analysis. High-purity DNA was isolated from whole blood using a DNA isolation kit (Tiangen, China) following the manufacturer's protocol. These works were approved by the ethics committee of Children's Hospital of Nanjing Medical University. The patients/participants provided their written informed consent to participate in this study.

### Targeted Next-Generation and Sanger Sequencing

Glycogen storage disease gene panel includes 37 genes. A list of the 37 genes is shown in Supplementary Table 1. All targeted regions including exons and exon-intron boundaries (plus 50 base pairs at each end) of 37 genes were captured using a GenCap kit (MyGenostics GenCap Enrichment technologies). The enrichment libraries were sequenced on Illumina HiSeq 2500 sequencer for paired read 150 bp. Sanger sequencing using ABI3130 DNA Analyser (Applied Biosystems, Foster City, CA, USA) was performed to confirm candidate variants.

### Bioinformatics Analysis

Data analysis was performed using a BWA-GATK procedure. After quality control, the clean reads were mapped to the UCSC hg19 human reference genome using BWA. Duplicated reads were removed using picard tools and mapping reads were used for variation detection. The variants of SNP and InDel were detected by GATK Haplotype Caller, then using GATK Variant Filtration to filter variant (8). Variations were annotated by ANNOVAR and associated with multiple databases and predicted by several functional prediction softwares (9). The nomenclature of all variations was referred to the NCBI reference sequence for *AGL (*NM000642, NM_000028*), G6PC (*NM_000151*), GAA (*NM_001079803, NM_000152*), GBE1 (*NM_000158*), PHKA2 (*NM_000292*), PHKB(*NM_000293*)*, and *SLC37A4(*NM_001164277*)*.

### Clinical and Laboratory Data

Clinical and biochemical data and long-term complications were retrieved from the paper and electronic files between April 2013 and November 2019. We recorded clinical disease parameters, including developmental status, signs of GI intolerance, and data of the feeding regimen. Height and weight were recorded at last check-up and compared with Chinese standard growth diagrams. Biochemical parameters included blood glucose, urine lactate, triglycerides, cholesterol, ALT, AST, imaging studies etc. Long-term complications were recorded at the last follow-up. Liver adenoma was manifested as one or more focal lesions of the liver.

Of the 32 patients, 84% were inpatients and 16% as outpatients. Due to the twice-changed computerized consultation system in our hospital, the laboratory results of some patients cannot be obtained, and some outpatients' parents only agreed to the panel-based sequencing and refused to perform additional tests. In order to inquire information not available in the hospital, we tried to contact parents by phone on follow-up.

### Statistical Analysis

Data were performed using SPSS 24.0 and Rstudio. Continuous data were presented as the mean and standard deviation (SD) or median. Frequency and percentage were used to report categorical variables. Associations of categorical variables were tested by the chi-square test or Fisher's exact test. All tests were two-sided and *P* < 0.05 was considered statistically significant.

## Results

### Demographic Features of Patients

Of the 32 patients with GSD, the median age at diagnosis was 5 months for GSD I, 15.5 months for GSD III, and 27 months for GSD IX. The average age of onset was 20.7 ± 13 months for the whole group (0–96 months). The male-to-female ratio was 20:12 (Table 1). Initial clinical presentation in most cases (87.1%) was in early childhood (≤3 years). Elevated liver transaminase level on a regular health checkup was the first symptom noticed for 34.1% of the patients, whereas hepatomegaly was observed in 84.4% of the patients. Two patients had mutations in the *GAA* gene corresponding to muscle GSD, whereas the other 30 patients had hepatic GSD. Of the 30 patients with hepatic GSD, 11 (37.9%) had GSD Ia, 2 (6.9%) had GSD Ib, 8 (24.1%) had GSD III, 1(3.4%) had GSD VI, 1(3.4%) had GSD IV, 6 (20.7%) had GSD IXa, and 1(3.4%) had GSD IXb. Table 1 summarizes the characterization of the genotypes. Compound heterozygous mutations were detected in 15 patients, homozygous mutations in 9 patients, hemizygous mutations in 5 patients, and heterozygous mutations in 3 patients.

**Table 1:** Ages (months) of onset of symptoms, mutations detected by panel sequencing, and clinical data of the patients with GSDs.

**Patients**	**Female**	**Months at presentation**	**Age at genetic diagnosis**	**Type**	**Gene**	**Mutation type**	**Initial clinicodemographic characterisitics**	**Maternal allele**	**Paternal allele**
1	Yes	44	45	Ia	*G6PC*	Compound-heterozygous	Failure to thrive, hepatomegaly, splenomegalia, hyperlipidemia,	c.238T>A	c.248 G>A
2		4	7	Ia	*G6PC*	Homozygous	Hepatomegaly, splenomegalia, fever, hypertransaminasemia, hyperuricemia, hyperlipidemia, anemia, fasting hypoglycemia, ketone positive	c.262_262delG	
3		1	4	Ia	*G6PC*	Compound-heterozygous	Hyperlipidemia	c.1022T>A	c.821C>T
4		4	7	Ia	*G6PC*	Compound-heterozygous	Fever, respiratory failure, septic shock, hyperlipidemia, fasting hypoglycemia, hypokalemia, ketone positive, kidney enlargement	c.262_262delG	c.353G>A
5		4.7	6.7	Ia	*G6PC*	Compound-heterozygous	Hepatomegaly, poor appetite, hyperlipidemia, anemia, fasting hypoglycemia	c.648G>T	c.262_262delG
6	Yes	3	23	Ia	*G6PC*	Compound-heterozygous	Hepatomegaly, hyperuricemia, hyperlipidemia, anemia, fasting hypoglycemia	c.518T>C	c.1022T>A
7		4	5	Ia	*G6PC*	Homozygous	Abdominal distension, hyperlipidemia, anemia, fasting hypoglycemia, ketone positive, high CK and LDH levels, Hypokalemia	c.248G>A	
8	Yes	13	15	Ia	*G6PC*	Homozygous	Growth retardation, repeated infection, hepatomegaly, splenomegalia, hyperuricemia, hyperlipidemia, anemia, fasting hypoglycemia, ketone positive	c.648G>T	c.648G>T
9	Yes	36	48	Ia	*G6PC*	Homozygous	Hepatomegaly, hypertransaminasemia, hyperuricemia, hyperlipidemia, fasting hypoglycemia, exercise intolerance, and muscle weakness	c.648G>T	c.648G>T
10	Yes	60	61	Ia	*G6PC*	Compound-heterozygous	High transaminase, hepatomegaly, hyperuricemia, hyperlipidemia, anemia, fasting hypoglycemia, ketone positive	c.113A>T	c.648G>T
11		96	97	Ia	*G6PC*	Homozygous	Hepatomegaly	c.648G>T	c.648G>T
12	Yes	0	10	Ib	*SLC37A4*	compound-heterozygous	abdominal distension, hepatomegaly, hyperuricemia, hyperlipidemia, anemia, atrial septal defect, septicemia, encephalopathy	c.572C>T	c.576_577insT
13	Yes	4	29	Ib	*SLC37A4*	Compound-heterozygous	Hepatomegaly, splenomegalia, hyperuricemia, hyperlipidemia, anemia, fasting hypoglycemia	g.5700_5703delAAGT	c.1042_1043delCT
14	Yes	36	37	II	*GAA*	Compound-heterozygous	Epilepsy, fever, myopathy, encephalopathy, myelitis, hypotonia	c.2237G>C	c.1935C>A
15		5	6.7	II	*GAA*	Compound-heterozygous	High transaminase, hepatomegaly, splenomegalia, hypertrophic cardiomyopathy	c.3214_3215insC	c.1316T>A
16		15	22	III	*AGL*	Compound-heterozygous	Hepatomegaly, hyperuricemia, hyperlipidemia, anemia, fasting hypoglycemia, hyperCKemia	c.1907C>G	c.3560-3561insA
17		12	27	III	*AGL*	Compound-heterozygous	Abdominal distension, hepatomegaly, hyperlipidemia, ketone positive	c.596A>T	c.607A>G
18	yes	19	33	III	*AGL*	Homozygous	High transaminase, hepatomegaly, hyperlipidemia, fasting hypoglycemia, ketone positive, high CK and LDH levels	c.3142_3158delTGTGGAGTAGGAAAATTinsAA	c.3142_3158delTGTGGAGTAGGAAAATTinsAA
19		1	3	III	*AGL*	Compound-heterozygous	Hepatomegaly, hyperlipidemia, glycogen storage in muscle, and liver	c.2207C>G	exons 6-7 del
20	Yes	13	14.7	III	*AGL*	Compound-heterozygous	Abdominal distension, hepatomegaly, splenomegalia, hyperlipidemia	c.2929C>T	c.1460G>C
21		19	48	IXa	*PHKA2*	Homozygous	Abdominal distension, hepatomegaly, splenomegalia, hyperlipidemia, fasting hypoglycemia, glycogen storage in liver	c.3341G>A	
22		11	14.5	IXa	*PHKA2*	Hemizygous	High transaminase, anemia	c.3471_ 3498delCAT CGGGGGCATCATCC ACGTGGACCAG	
23		36	37.5	IXa	*PHKA2*	Hemizygous	Hepatomegaly, high transaminase, anemia, hyperlipidemia	c.739C>T	
24		36	38	IXa	*PHKA2*	Hemizygous	High transaminase, hepatomegaly, splenomegalia,	exons 6-9 dup	
25		36	37	IXa	*PHKA2*	Hemizygous	High transaminase, hepatomegaly, glycogen storage in liver	c.3210_3212delGAG	
26		0	2.5	IXa	*PHKA2*	Hemizygous	Jaundice, hepatomegaly, splenomegalia, anemia	c.2272G>A	
27		24	26	IXb	*PHKB*	Homozygous	High transaminase	c.668_669insAGGA	c.668_669insAGGA
28		12	24	IV	*GBE1*	Compound-heterozygous	High transaminase, anemia,	c.610G>T	c.950G>A
29		27	31	VI	*PYGL*	Homozygous	High transaminase, hyperlipidemia, ketone positive	m.2681_2678delATTCinsTTG	m.2681_2678delATTCinsTTG
30	Yes	32	33	III	*AGL*	Heterozygous	Fever, hyperlipidemia, fasting hypoglycemia, sepsis, hypertransaminasemia, coagulopathy	_	c.1G>A
31	Yes	1	5.5	III	*AGL*	Heterozygous	Jaundice, high transaminase	c.188G>A	_
32		54	60	III	*AGL*	Heterozygous	High transaminase, hepatomegaly, hyperlipidemia, anemia		c.1444A>G

### Laboratory Findings

**Figure 1:**
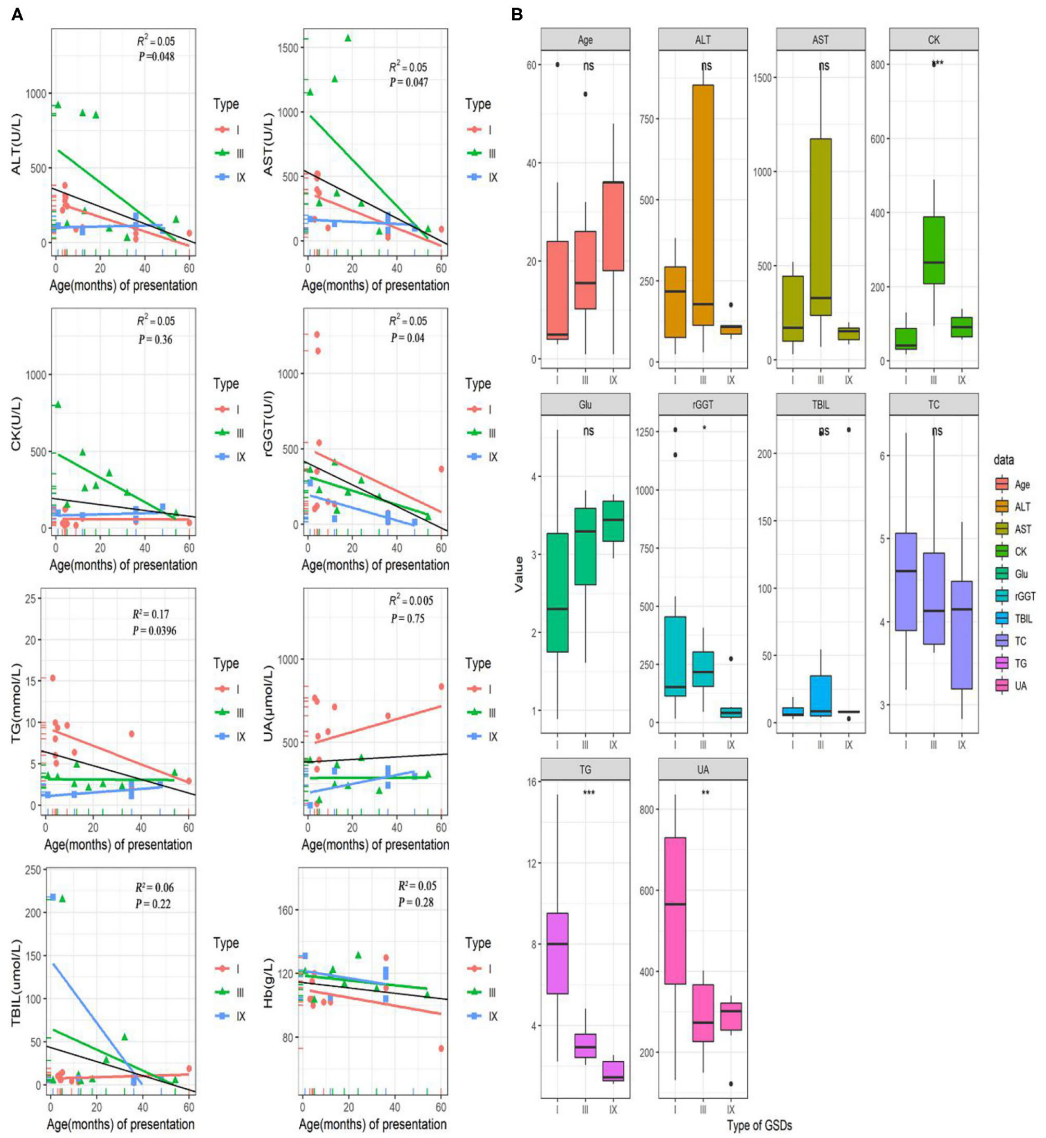
Laboratory test and data for GSD patients. **(A)** Correlation of AST, ALT, rGGT, TG, CK, UA, TBIL, and Hb levels with age (months) at onset in pediatric patients with GSDs. The colorful lines represent fitting curve for GSD I(red), III(green), and IX(blue). The black line is the regression line for all GSD patients. **(B)** Differences in several laboratory inspection items between 11 GSD I, eight GSD III, and seven GSD IX. Statistical method is one-way analysis of variance. ^⋆^*P* < 0.05; ^⋆⋆^*P* < 0.01; ^⋆⋆⋆^*P* < 0.001. ALT, alanine aminotransferase; AST, aspartate aminotransferases; CK, creatine kinase; GGT, gamma-glutamyl transferase; TG, triglyceride; UA, uric acid; TBIL, total bilirubin; Hb, hemoglobin; TC, cholesterol; Glu, blood glucose.

**Figure 2:**
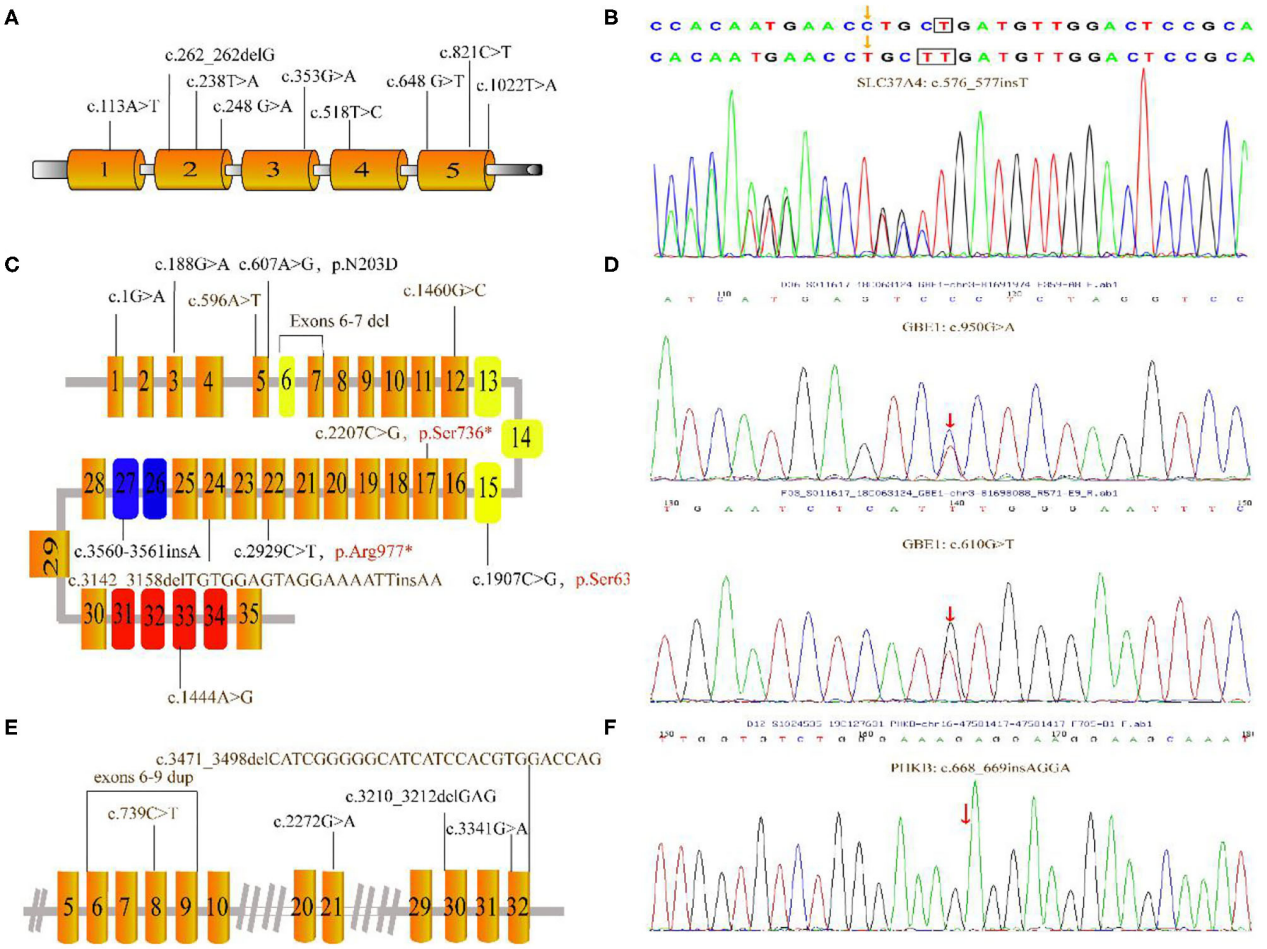
Mutations depicted in this research. **(A)** Schematic diagram of the *G6PC* gene showed the locations of the mutations. **(B)** The c.576_577insT mutation in *SLC37A* identified by GSD-NGS panel(red arrow). **(C)** Organization of the *AGL* gene and mutations associated this study; the three main functional domains including transferase catalytic residues (yellow boxes), amylo-1, 6-glucosidase catalytic site (blue boxes) and glycogen-binding domain (red boxes). **(D)** Partial genomic sequences of the *GBE1* gene with heterozygous missense mutations (red arrow). **(E)** Partial schematic representation of the *PHKA2* gene. **(F)** Electropherogram of PHKB cDNA of our patient. The number in each box represent exons. Unreported mutations are denoted in brown, and reported mutations are in black. Mutation marked in red stands for nonsense mutation. Gray lines indicate introns (not to scale).

The laboratory test results were available for 25/32(78%) patients (including 11 of GSD I, 8 of GSD III, 6 of GSD IX; in Figure 1). In this study, the serum aspartate aminotransferase (AST), alanine transaminase (ALT), gamma-glutamyl transferase (GGT), and triglyceride (TG) concentrations tended to be high in the early ages and correlated negatively with age (*P* < 0.05). The median (IQR) for AST, ALT, GGT and TG concentrations were 170 (1566.7) U/L, 120 (916) U/L, 152 (1259) U/L and 3.34 (15.36) mmol/L, respectively. Unlike AST, ALT, GGT and TG, creatine kinase (CK), uric acid (UA), total bilirubin (TBIL), and hemoglobin (Hb) levels did not differ significantly with age (*P* > 0.05; Figure 2A). Age, AST, ALT, glucose, TBIL and TC levels did not differ between GSD I, III and IX, groups (*P* > 0.05). In the GSD III group, the mean serum CK concentration was 331 U/L vs. 59 U/L in the GSD I group and 93U/L in the GSD IX group (*P* < 0.001). The mean serum GGT concentration was 389 U/L in the GSD I group vs. 224 U/L in GSD III and 76 U/L in GSD IX (*P* < 0.05). The mean serum TG concentration differed significantly different between groups (7.6 mmol/L in GSD I vs. 3.1 mmol/L in GSD III, vs. 1.7 mmol/L in GSD IX; *P* < 0.001). The mean serum UA level in the GSD I group (548 umol/L) was significantly higher than in the GSD III (286 μmol/L) and GSD IX (272 μmol/L) groups (*P* < 0.01).

### Gene and phenotypes

Overall, Figure 2 presents the 41 different variants, including 24 missense variants (58.5%), one synonymous variant (2.4%), three non-sense variants (7.3%), one splice variant (2.4%), four frameshift variants (9.8%), one deletion variant (2.4%), four insertions (9.8%), two deletion-insertions(4.9%) and one duplication (3%) variant. In our present study, both pathogenic variants were identified in 29 infants confirming the diagnosis of GSDs, while one allelic variant was identified in three patients. All mothers were asymptomatic. All identified variants from the patients were confirmed using polymerase chain reaction–based Sanger sequencing method and inherited from their carrier parents.

### GSD Ia

Nine variants were detected in the *G6PC* gene of 11 patients, all of which have been reported before. These reported variants include one nonsense change (c.648G>T), one frameshift variant (c.262_262delG) and seven missense variant (c.113A>T; c.238T>A; c.248G>A; c.353G>A; c.518T>C; c.821C>T and c.1022T>A). Among those, the majority (36.4%) were the common synonymous variant c.648G>T, followed by the frameshift variant c.262_262delG (13.6%). Clinical and biochemical parameters were significant different among these patients with GSD 1a. Almost all patients had hepatomegaly and hypoglycemia. Anemia was observed in 6 patients with GSD 1a especially for P10. Other major biochemical parameters were elevated liver transaminase levels, hyperlipidemia, and hyperuricemia (5 patients; 45%).

### GSD Ib

In two patients, three known pathogenic variants in *SLC37A4* were detected. These variants include one missense variant c.572C>T, one splicing variant g.5700_5703delAAGT and one frameshift variant c.1042_1043delCT. In addition, patient 13 shared a novel c.576_577insT variant, located at the fifth SLC37A4 exon. However, she suffered from nausea, recurrent infectious with otitis, diarrhea, fatigue, poor feeding, elevated TG, hypoglycemia and hepatomegaly at the first year of life.

### GSD II

*GAA* gene variant was performed in 2 patients, and sequencing results from patient 14 showed the variant of c.2237G>C at exon 17 was inherited from the mother, and the variant of c.1935C>A at exon 14 was inherited from the father. Therefore, the index patient has a compound heterozygous genotype with the known variant c.2237G>C in exon 17 and c.1935C>A in exon 14. In patient 15, the sequencing panel revealed compound heterozygous c. [1316T>A; c.3214_3215insC] genotype. These two patients had different clinical presentations. Patient P14, a 3-year-old girl referred to our hospital with severe cerebral meningitis, convulsion, pneumonia, respiratory failure, and abnormal hepatic function, whereas Patient 15, a 6-month-boy had hepatosplenomegaly, hypertrophic cardiomyopathy, and abnormal laboratory examination including increased transaminase (ALT 142U/L, AST 217U/L) and CK level (681U/L).

### GSD III

Each of the eight patients had a different *AGL* variant. Among the 12 mutations, six were known variants—c.1G>A; c.188G>A; c.607A>G; c.3560_3561insA; c.2929C>T; c.1907C>G—and six were novel variants (c.1444A>G; c.596A>T; c.3142_3158delTGTGGAGTAGGAAAATTinsAA; c.2207C>G; exons 6-7 del; c.1460G>C). In 3 patients, only a single mutation on 1 allele was identified. P19 with the novel variants c. [2207C>G] and exons 6-7 deletion showed hepatomegaly in the first month of life. Laboratory examinations showed elevated liver transaminases but TG concentration and UA were normal to slightly increased. Liver biopsy showed steatosis. The liver was enlarged with normal echogenicity.

### GSD IV

NGS panel analysis revealed compound heterozygous missense mutations c.610G>T [p.V204L, located in exon 5] and c.950G>A [p.G317E, located in exon 7] in the patient. In addition, the former mutation was detected in his mother and the latter in his father; while both of these were heterozygous mutations and not listed in the Human Gene Mutation Database (HGMD). These two novel variants was identified in the Patient 28, who suffered from suffered from slightly increased liver transaminases, whereas without hepatosplenomegaly, growth retardation, and any neurologic symptoms.

### GSD IX

We found 6 mutations of the *PHKA2* gene in 6 patients with GSD IXa, including 3 missense mutations (c.3341G>A; c.739C>T; c.2272G>A), 2 frameshift mutations (c.3471_c.3498delCATCGGGGGCATCATCCACGTGGACCAG; c.3210_3212delGAG), and 1 repeat mutation (exon6-9). Four of these were novel. In our patients with GSD IXa, all 3 patients with these novel mutations (c.739C>T; exon6-9 rep; c.3471_3498delCATCGGGGGCATCATCCACGTGGACCAG) showed hepatomegaly, elevated liver transaminases, and slightly decreased blood glucose. Besides, the only patient with GSD IXb had the frameshift mutation (c.668_669insAGGA) of the *PHKB* gene which was not reported in the HGMD. This 2-year-old boy had suffered from mild hepatosplenomegaly and increased liver transaminases with normal fasting blood glucose, UA, and TG level.

### Follow-Up

In totality, 17 patients were followed up, and the median follow-up age was 36 months (1–96 months). Among these patients, 5 had GSD Ia, 2 had GSD Ib, 2 had GSD II, 2 had GSD III, 4 had GSD IXa, 1 each had GSD IV and GSD IXb.

Weight and height data available for 12 patients. Patient 20 with GSD III had returned to regular weight gain but developed a short stature (height <2 standard deviations less than the mean) on an uncooked cornstarch diet despite increased dose. In addition, 3 patients with GSD I (patients 2, 8, 13) had heights below the 10th percentile for their age. Only Patient 24 with GSD IXa was gradually becoming obese; none had a weight below the 10th percentile for their age.

Treatment strategies varied considerably between patients; 13 patients initially received uncooked cornstarch after diagnosis, 3 of these patients also received protein supplementation, and 1 of them received high amylose starch subsequently. Four patients discontinued uncooked cornstarch after 1 year of treatment, and 2 patients consumed it only once a night. In total 4 patients had received no dietary treatment at all. Although blood glucose and ketone levels before food intake and after exercise is necessary to determine the intake level for uncooked cornstarch and other dietary inclusions, many parents fail regular monitoring owing to the high cost and effort.

When patients who underwent panel-based NGS were asked to recall the frequency of hypoglycemia episodes after diagnosis, patient 13 with GSD Ib had frequent episodes of hypoglycemia with convulsions and needed hospitalization, 1 patient (Patient 2) with GSD Ia had 1-2 hypoglycemic episodes per week, 3 patients with GSD Ia had 2-3 episodes per month, 1 patient (Patient 24) with GSD IXa had 1 or 2 episodes per year. Other patients treated with uncooked cornstarch had stable blood glucose levels without hypoglycemia episodes.

Liver adenoma was reported in 4 patients (including 2 patients with GSD III and 2 patients with GSD IX). Liver enzymes returned to normal in 2 patients with GSD Ia with treatment. The patient with GSD Ib experienced enteritis with watery stool 2–3 times a day. Neuromuscular complications were observed in 3 cases (Patient 12 with GSD Ib, Patient 17 with GSD III, and Patient 25 with GSD IXa) with functional features including fatigability, weakness, and delayed walking. With regard to variability in individual clinical manifestations, follow-up data, and treatment regimens, the effects of treatment on parameters such as growth, complications, and long-term prognosis could not be determined.

## Discussion

This study summarized the clinical data and long-term treatment outcomes of 32 patients with 6 types of GSDs and 10 novel mutations in the *AGL, SLC37A, GBE1, PHKB*, and *PHKA2* genes. Our results show that the NGS panel was effective in genetic diagnosis in clinical settings, particularly for patients with mild phenotypes. Specific dietary therapies can alleviate clinical symptoms and biochemical abnormalities in patients of GSDs (10). In our study, all 4 patients with liver adenoma received no dietary treatment, whereas other patients underwent dietary therapy. Our results show that with appropriate dietary therapy, liver adenoma could be avoided. However, 10 of 17 patients in our cohort did not strictly follow diet management, whereas 9 patients had complications on their last follow-up, which signifies the importance of strict diet monitoring and regular follow-up in GSD patients.

In our patient cohort, GSD-I was the most common subtype (40.6%), which is further classified into 1a and 1b, which are caused by a mutation in *G6PC* and *SLC37A4* genes, respectively. Subsequently, the GSD-NGS panel revealed 8 recurrent mutations in 11 patients with GSD Ia, and 1 novel mutation (c.576_577insT) and 3 reported mutations in 2 patients with GSD Ib. The reported splice variant in exon 5 (c.648G > T) of the G6PC gene was the most common mutation (8 of 22 alleles, 36.4%) among our Chinese patients with GSD Ia, which is in contrast with observations in a Korean cohort (81 of 94 alleles, 86.2%) and in a Japanese cohort (88 of 102 alleles, 86.4%) (11). Early symptoms and signs at first admission include severe intolerance to fasting, growth retardation, and hepatomegaly.

Although diet therapy greatly improves the life expectancy of patients with GSD I, our parents' guardians seemed unsuccessful in strictly managing their children's diet, leading to their diet being poorly controlled. In our study, 7 children (5 with type Ia and 2 with type Ib) were followed up for an 55 months (4–96 months). Four patients had unstable blood glucose levels, 4 had rhinitis (about once a week), and 2 frequently had a cold. Patient 9 with GSD Ia had kidney stones, and Patient 13 with GSD Ib showed muscle involvement and enteritis. However, other important long-term complications including hepatocellular tumors, proteinuria, renal insufficiency, and osteoporosis were not been observed in our patients.

GSDIII caused by a deficiency in glycogen debranching enzyme (GDE) encoded by the AGL gene that is which located on chromosome 1p21, is the second most frequent cause of glycogenosis. In this study, we analyzed 8 Chinese patients with GSD III and identified 12 different mutations, 6 of which were not previously reported. One of the unreported mutations (exons 6-7) is deletion, whereas the others were 3 missense mutations (c.596A>T; c.1460G>C; c.1444A>G), a nonsense mutation (c.2207C>G, p.Ser736^*^) resulting in a truncated protein, an insertion mutation (c.3560-3561insA) and a deletion-insertion mutation (p.Cys1048_Phe1053delinsN). Patient 20 with the nonsense variant (c.2929C>T, p.Arg977^*^) had severe hepatomegaly (extending 10 cm below the costal margin along the midioclavicular-line) and hyperlipidemia at first presentation. The liver returned to its normal size after 19 months of cornstarch treatment (4 times a day). However, given the small sample size, no statistically significant correlation could be established between the AGL genotype and the occurrence of complications in our patients. Only few studies on neuromuscular involvement in patients with GSD III have been reported. Although myopathic changes often occur in older age and are correlated with high CK levels (12), early muscle disease cannot be ruled out even in case of normal CK concentrations. Patient 17, who had 2 missense mutations c.[596A>T];[607A>G], had hepatomegaly, abnormal liver function, and normal serum CK levels in the early stages of onset; however, electromyography (EMG) indicated a low CMAP amplitude of the right common peroneal nerve with myogenic damage, and full abdominal computed tomography imaging showed hypoechoic lesions at 32 months of follow-up. Because skeletal muscle involvement occurs with age (13), some patients did not have muscle involvement during the study or follow-up; hence, we could not ascertain the molecular subtype of GSD III or if patients were in the pre-symptomatic stage. Although some children showed relatively mild manifestations in the early stages of the disease, regular use of EMG, and echocardiography along with a strict dietary regimen that ensures high levels of protein, energy, and complex sugars to prevent serious complications. However, patients 29, 30, and 31 who had high liver transaminase and hepatomegaly had only one mutation in the *AGL* gene instead of 2. The hidden mutations are likely in the regulatory regions that are not covered by the GSD-NGS panel, given the AGL gene is one of the largest genes (36 exons) and coves the most gaps.

GSD IX results from phosphorylase kinase (PhK) deficiency. The most common GSD IX subtype is IXa that occurs because of mutations in the PHKA2 gene located in Xp22.2–22.1. In this report, we identified 3 missense mutations, 3 frameshift mutations, and 1 repetitive mutation; 4 of them were novel. In addition, 2 mutations in our study were pathogenic, 2 variants were likely pathogenic, and 3 mutations were uncertain according to American College of Medical Genetics' guidelines. Although our study highlights the heterogeneity of genetic mutations among our patient cohort, we could not determine the correlation between the genotype and phenotype for GSD IX given the small sample size. In agreement with previous reports (14), 85.7% of our patients with GSD IX had hepatomegaly, 28.6% had elevated blood lipids level, 71.4% showed increased liver transaminase level, and 100% showed fasting hypoglycemia, which was not detected at admission. In addition, normoglycemic ketonemia was common among our patients with GSD IX. As GSD IXa often clinically overlaps with other GSD types, it is mainly identified using genetic tests (15). The long-term outcomes of GSD IX have been rarely reported. In our study, the outcomes of frequent daytime feeding of uncooked cornstarch were recorded in 6 patients with GSD IX and the longest follow-up on treatment was 66 months. Patient 23 with GSD IXa had obesity, and Patient 27 with GSD IXb had normal alanine aminotransferase but elevated CK level. Furthermore, Patient 24 developed hypoglycemia, asthenia, and fatigue after exercise while on treatment. The clinical manifestations of the disease resolve with age, and the patient can become asymptomatic. In the end, as treatment is usually not required, GSD is described as a benign condition (16). However, the clinical presentation is heterogeneous and variable, ranging from mild (hepatomegaly and elevated transaminase levels) to severe (hypoglycemia, short stature, progressive liver disease or cirrhosis) (17). Although no consensus on treatment for patients with GSD IX existed, further controlled studies following up on the benefits of therapeutic interventions such as uncooked cornstarch diet are necessary.

Our study showed a decrease in aminotransferase levels with age although it did not normalize in most patients. In this model, hyperuricemia was present in one-fifth of our patients. Twenty-two patients had high TG levels, which was found to correlate negatively with age (*P* < 0.05). Moreover, serum ALT, AST, rGGT levels and age at admission were negatively correlated. Based on the laboratory data, GSD III is more likely to present with elevated CK levels than GSD I and GSD IX. An elevated CK level is considered a possible serological marker of GSD III (18). Patients with GSDI had higher serum TG, UA, and rGGT levels than patients with GSDIII and GSD IX.

GSD II, known as Pompe disease, is caused by a defiency in the lysosomal acid-α-glucosidase (GAA). GSD II has an incidence of 1 per 40,000 births. One of the patients in our study had the compound heterozygous c. [1316T>A];[3214_3215insC] mutation of the GAA gene, which led to the infantile-onset Pompe disease with hypertrophic cardiomyopathy. Another patient who had late-onset GSD II had compound heterozygous c.[2237G>C];[1935C>A] mutation of the GAA gene, which manifested severe pneumonia and respiratory failure, necessitating invasive ventilation, acute disseminated encephalomyelitis, elevated transaminase levels, and progressive limb muscle weakness at onset age of 36 months. Parents of these patients did not have a consanguineous marriage, the mothers had normal pregnancies and deliveries. Once GSD II patients develop pneumonia and respiratory failure requiring mechanical ventilation, withdrawal of mechanical ventilation is difficult (19). As the number of patients with GSD II in our study was small, we could not conclusively establish correlations between the genotype and phenotype.

GSD IV is a rare autosomal recessive metabolic disorder caused by *GBE1* mutations and accounts for <1% of GSDs. Only 1 patient had GSD IV in our study. On admission, he had isolated elevation of serum transaminase levels (AST 312U/L, ALT 129U/L), without hepatosplenomegaly, growth retardation, and any neurologic symptoms. He did not fulfill clinical or laboratory criteria of GSD IV for several years, and the diagnosis was established serendipitously through a GSD-NGS panel assay. This case illustrates the value of GSD-NGS panel assays for early diagnosis in mild phenotypes, particularly in the context of clinical heterogeneity. GSD VI is the result of defect in the *PYGL* gene expression, which is located on chromosome 14q21-q22. Patients with GSD VI typically experience growth retardation, hepatomegaly, and mild hypoglycemia (20). Patient 29 was presented with poor weight and height gain since infancy, whereas mild hypoglycemia and hepatic transaminase level elevation were seen at the age of 2 years and 3 months when the patient was hospitalized for a fracture. Although GSD VI was previously believed to be a benign disease, recent reports suggest that patients with GSD VI can develop with liver fibrosis and hepatocellular carcinoma (21). Hence, long-term monitoring of the hepatic status in such patients is necessary.

Varying manifestations and inaccessibility to genetic testing could be the reasons why GSDs remain undiagnosed in most patients. Currently, targeted panel-based NGS is a useful addition to a detailed clinical workup for pediatric genetic diseases in case of strong clinical suspicion, and well-known candidate genes lists (22, 23). We chose targeted NGS to avoid expensive full-gene Sanger sequencing and numerous mutations generated using whole exome sequencing. Observations of this study support the use of the GSD-NGS panel as an effective tool to identifying GSD-associated pathogenicity and novel mutations in patients exhibiting typical and related phenotypes with or without family history.

Strengths of our study are that it is a retrospective study of clinical and laboratory parameters of children with multiple GSD types caused by different gene mutations, and we were able to avoid invasive liver puncture in some patients with mild symptoms for definitive diagnosis. We could identify all gene mutations by owing to easy availability of the GSD-NGS panel assay, which has guiding value for accurate prognostication and treatment. Our study has limitations. First, patients in our research cohort were relatively young at enrolment, and we may have missed some biochemical test results performed outside our hospital. In addition, follow-up data for clinical and biochemical assays at certain time points were missing because of loss of contact with some patients. Particularly, data regarding dietary treatment was extremely limited. Thus, limitations might may have caused underrepresentation, limiting our knowledge of the progression in these patients with GSD.

## Conclusions

In summary, we have demonstrated the practicability of this panel-based NGS, able to detect the variants of patients with GSD, succeed in averting the invasive liver puncture in mild phenotypes. Hepatic GSDs mainly manifested as fasting hypoglycemia, hepatomegaly, hyperlipidemia, and poor growth, whereas, the laboratory results and prognosis of each type are different. Patients in the group GSD III had higher serum CK concentration compared with patients in the GSD I and GSD IX group. In addition, serum rGGT, TG and UA concentration were higher in patients with GSD I than those in patients with GSD III and GSD IX. Meanwhile, the uncooked cornstarch is very important for the treatment of hepatic GSDs. Although some patients had mild symptoms at the first visit or improved symptoms with age, we need to further follow up with these patients and study personalized treatment, as well as the possible benefits of strict patient management on the uncooked cornstarch diet.

## Data Availability Statement

The datasets presented in this study can be found in online repositories. The names of the repository/repositories and accession number(s) can be found below: GenBank MW211012 - MW211111.

## Ethics Statement

The patients/participants provided their written informed consent to participate in this study.

## Author Contributions

SY and ZZ are responsible for the provision of the overall idea and writing articles. ZY, JY, and LQ collects data and modifies the paper. ZB is responsible for genetic sequencing analysis and prepared tables. LZ and CW conceived and reviewed the drafts of the paper. All authors read and approved the final manuscript.

## Conflict of Interest

The authors declare that the research was conducted in the absence of any commercial or financial relationships that could be construed as a potential conflict of interest.

## Abbreviations

NGS: next-generation sequencing
GSD: Glycogen storage disease
AGL: amylo-alpha-1, 6-glucosidase
G6PC: glucose-6-phosphatase-alpha
GAA: acid alpha-glucosidase
*GBE1*: glycogen branching enzyme
*PHKA2*: phosphorylase kinase α-subunits
*PHKB*: phosphorylase kinase β-subunits
*SLC37A4*: glucose-6-phosphatase transporter
ALT: alanine aminotransferase
AST: aspartate aminotransferases
GGT: gamma-glutamyl transferase
TG: triglyceride
UA: uric acid
TBIL: total bilirubin
CK: creatine kinase
Hb: hemoglobin
HGMD: Human Gene Mutation Database.
